# PTTG1 regulated by miR-146a-3p promotes bladder cancer migration, invasion, metastasis and growth

**DOI:** 10.18632/oncotarget.13507

**Published:** 2016-11-22

**Authors:** Wei Xiang, Xinchao Wu, Chao Huang, Miao Wang, Xian Zhao, Gang Luo, Yawei Li, Guosong Jiang, Xingyuan Xiao, Fuqing Zeng

**Affiliations:** ^1^ Department of Urology, Union Hospital, Tongji Medical College, Huazhong University of Science and Technology, Wuhan 430022, Hubei Province, PR China; ^2^ Department of Urology, Wuhan No.1 Hospital, Tongji Medical College, Huazhong University of Science and Technology, Wuhan 430022, Hubei Province, PR China

**Keywords:** bladder cancer, PTTG1, miR-146a-3p

## Abstract

Pituitary tumor-transforming gene 1 (PTTG1) is identified as an oncogene, and overexpresses in many tumors. However, the role of PTTG1 in bladder cancer (BC) hasn't yet been characterized well. In this study, we showed the expression of PTTG1 mRNA and protein were both significantly increased in BC tissues and cells. The PTTG1 protein levels were positive correlated with increased tumor size, tumor–node–metastasis (TNM) stage, lymphatic invasion and distant metastasis of BC. PTTG1 knockdown dramatically suppressed the migration, invasion, metastasis and growth, and induced senescence and cell-cycle arrest at G0/G1 phase of BC cells. We further identified PTTG1 was the direct target of miR-146a-3p through using target prediction algorithms and luciferase reporter assay. miR-146a-3p was low expressed and negatively correlated with PTTG1 levels in BC tissues and cells. miR-146a-3p overexpression inhibited migration, invasion, metastasis and growth, and induced senescence of BC cells. Rescue experiment suggested ectopic expression of miR-146a-3p and PTTG1 suppressed migration, invasion and induced cell cycle arrest and senescence of BC cells compared to PTTG1 overexpression, confirming miR-146a-3p inhibited BC progression by targeting PTTG1. In summary, our study found miR-146a-3p/PTTG1 axis regulated BC migration, invasion, metastasis and growth, and might be a targets for BC therapy.

## INTRODUCTION

BC is a common malignancy and the second most lethal type of tumor among individuals with genitourinary cancer in the west world [1]. The five-year recurrence rate of patients with early stage BC is over 50% after endoscope surgery, and the prognosis of BC remains poor [2]. Hence, it is of great significance to seek novel therapeutic targets and approaches.

PTTG1 which resides at human chromosome 5q33.3 and encodes a securin protein, is first isolated from rat pituitary tumor cells in 1997 [3]. As an anaphase inhibitor, PTTG1 inhibits the separase activity and prevents the separation of premature chromosome, and therefore is involved in several vital cellular events [4, 5]. PTTG1 was subsequently demonstrated to be abundantly expressed in several types of human malignancies, such as pituitary, pulmonary, pancreatic, renal, prostatic and colorectal cancers [6–11]. Previous studies indicated that PTTG1 is involved in the modulation of metastasis in human ovarian cancer [12], and is implicated in the regulation of senescence and growth of pituitary tumor [13]. PTTG1 was also found to enhance the migration and invasion of breast cancer cells [14]. Notably, PTTG1 was found to promote the resistance to gefitinib-induced apoptosis in human BC cell lines, implying a possible role for PTTG1 in BC [15]. Nonetheless, the precise functions of PTTG1 in BC have yet to be elucidated. The mechanisms underlying the regulation and the downstream signaling pathway of PTTG1 in various cancer cells remain largely unknown.

Genetic and epigenetic changes, like polymorphism, mutation, loss of heterozygosity (LOH) and DNA methylation are believed to be implicated in the dysfunction of human genes [16–19]. However, Kanakis D et al. [20] failed to find any mutations in the main promoter region of PTTG1 which may result in the overexpression of the PTTG1. Another study on the genetic changes of PTTG1 also excluded methylation and LOH as causes of PTTG1 aberration in human cancers [21]. miRNAs are small non-coding RNA which participate in a wide array of cellular processes [22]. Meanwhile, miRNAs are confirmed to play a pivotal role in the development and progression of human cancers [23]. Noticeably, previous studies discovered the aberrant expression of miRNAs in BC cells [24]. Therefore, we sought to determine whether miRNAs modulate PTTG1 in BC. By employing the prediction software packages of target gene, PTTG1 might be a target of miR-146a-3p.

In the present study, we first investigated the role of PTTG1 in growth, migration, invasion and metastasis of BC. Then, we explored the regulation mechanism of PTTG1 by miR-146a-3p and the role of miR-146a-3p in BC growth, migration, invasion and metastasis.

## RESULTS

### PTTG1 expression is upregulated in BC tissues and cells

**Figure 1:**
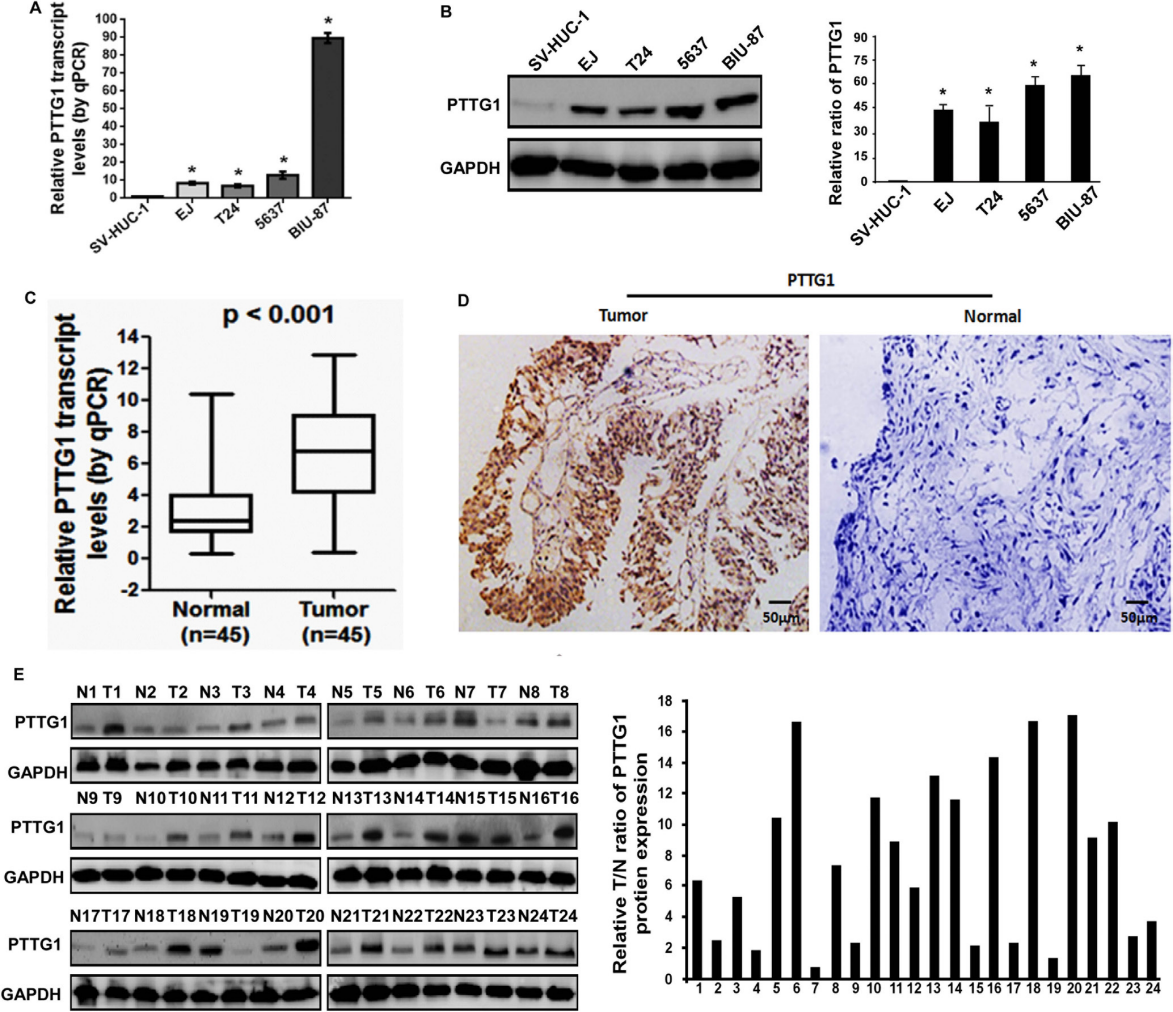
PTTG1 was overexpressed in BC tissues and cell lines (**A**) Real-time RT-PCR analysis suggested PTTG1 was upregulated in BC cells compared to the normal uroepithelium cell SV-HUC-1. (**B**) Western blot assay suggested PTTG1 was upregulated in BC cells compared to the normal uroepithelium cell SV-HUC-1. GAPDH was used as the loading control. (**C**) Real-time RT-PCR analysis suggested PTTG1 was upregulated in BC tissues compared to the adjacent normal bladder tissues. (**D**) IHC assay suggested PTTG1 was upregulated in BC tissues compared to the adjacent normal bladder tissues. Scale bar, 50 μm. (**E**) Western blot assay suggested PTTG1 was upregulated in BC tissues compared to the adjacent normal bladder tissues. GAPDH was used as the loading control. *P < 0.05. Results were the means ± SD in triplicate.

To determine the role of PTTG1 in BC progression, we first analyzed PTTG1 expression in BC tissues and cells. Real-time RT-PCR and western blot analysis suggested PTTG1 was significantly upregulated in BC cells compared to the normal uroepithelium cell SV-HUC-1 (Figure 1A and 1B). We also analyzed PTTG1 levels in the 45 pairs of bladder cancer tissues and adjacent normal bladder tissues. Real-time RT-PCR suggested PTTG1 was significantly upregulated in bladder cancer tissues compared to adjacent normal bladder tissues (Figure 1C). IHC analysis suggested PTTG1 was localized in the cell cytoplasm of BC cells, and was strikingly increased in BC tissues (Figure 1D). Western blot analysis also suggested PTTG1 was upregulated in bladder cancer tissues compared to the normal bladder tissues (Figure 1E). Moreover, we also analyzed the correlation between PTTG1 expression and some clinical parameters, such as gender, age, tumor size, histologic grade, TNM stage, lymphatic invasion and distant metastasis, and found increased PTTG1 expression was correlated with tumor size, TNM stage, lymphatic invasion and distant metastasis, but there was no significant correlation between PTTG1 with other clinical pathologic characteristics including gender, age, or histologic grade in BC (Table 1).

**Table 1:** Correlation between PTTG1 protein level and clinicopathologic characteristics of BC

Parameters	Number of cases	PTTG1 protein level		*P* value
		Low	High	
Gender				
Male	28	5	23	0.6447
Female	17	4	13	
Age (y)				
< 55	14	3	11	0.8721
> 55	31	6	25	
Tumor size, cm				
< 3	22	8	14	0.0073^a^
≥ 3	23	1	22	
Histologic grade High/moderate	24	7	17	0.1003
Poor	21	2	19	
TNM stage				
I, II	23	8	15	0.0112^a^
III, IV	22	1	21	
Lymphatic invasion				
Negative	29	9	20	0.0127^a^
Positive	16	0	16	
Distant metastasis				
Negative	31	9	22	0.0242^a^
Positive	14	0	14	

a *P* < 0.05.

### Knockdown of PTTG1 inhibits the migration, invasion, metastasis and growth, and induces senescence of BC cells

**Figure 2:**
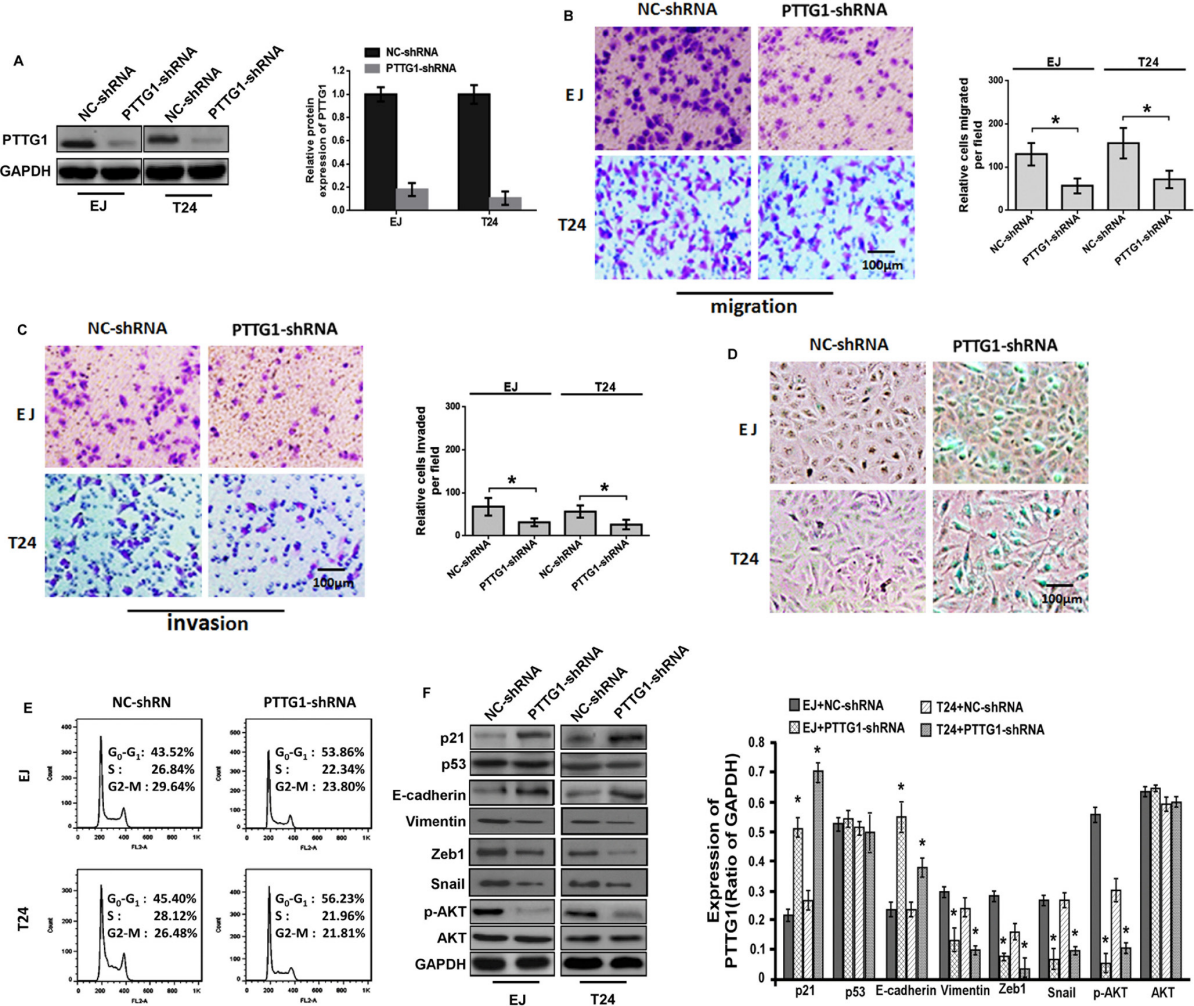
PTTG1 knockdown inhibited migration and invasion and induced cell cycle arrest and senescence (**A**) Western blot assay suggested shRNA of PTTG1 downregulated PTTG1 expression. GAPDH was used as the loading control. (**B**) Transwell assay without Matrigel suggested PTTG1 knockdown inhibited cell migration. Scale bar, 100 μm. (**C**) Transwell assay with Matrigel suggested PTTG1 knockdown inhibited cell invasion. Scale bar, 100 μm. (**D**) SA-βgal activity assay suggested PTTG1 knockdown induced BC cell senescence. Scale bar, 100 μm. (**E**) Flow cytometry assay suggested PTTG1 knockdown arrested cell cycle in G0/G1 phase. (**F**) Western blot assay studied p21, p53, E-cadherin, Vimentin, Zeb1, Snail, p-AKT and AKT expression after PTTG1 knockdown in BC cells, GAPDH was used as the loading control. *P < 0.05. Results were the means ± SD in triplicate.

To unravel the function of PTTG1 in oncogenesis of BC, PTTG1 was significantly downregulated in EJ and T24 cells by shRNA for PTTG1 (PTTG1-shRNA) (Figure 2A). Transwell analysis without Matrigel suggested PTTG1 knockdown significantly inhibited BC cell migration (Figure 2B). Transwell analysis with Matrigel suggested PTTG1 knockdown significantly inhibited BC cell invasion (Figure 2C). We also determined the role of PTTG1 in BC cell proliferation and senescence. Beta-galactosidase (SA-βgal) activity assay suggested PTTG1 knockdown induced a significant increase of senescent cells in both EJ and T24 cells as compared to the control groups (Figure 2D). Flow cytometry assay suggested PTTG1 knockdown arrested cell cycle in G0/G1 phase compared to the control groups (Figure 2E). These findings suggested PTTG1 knockdown inhibited BC cell migration, invasion and cell cycle progression, and induced senescence.

To explore the regulatory mechanism of PTTG1, we examined some key proteins that might be involved in epithelial–mesenchymal transition (EMT), senescence, migration, invasion and metastasis. As shown in Figure 2F, western blot assay showed that when PTTG1 was downregulated, the levels of p21, E-cadherin and the phosphorylation of AKT (p-AKT, one activated form of AKT) were significantly increased, the levels of Vimentin, Zeb1 and Snail were significantly downregulated, however, AKT and p53 protein didn't show significant change, suggesting PTTG1 could regulate senescence, migration, invasion and metastasis associated proteins.

**Figure 3:**
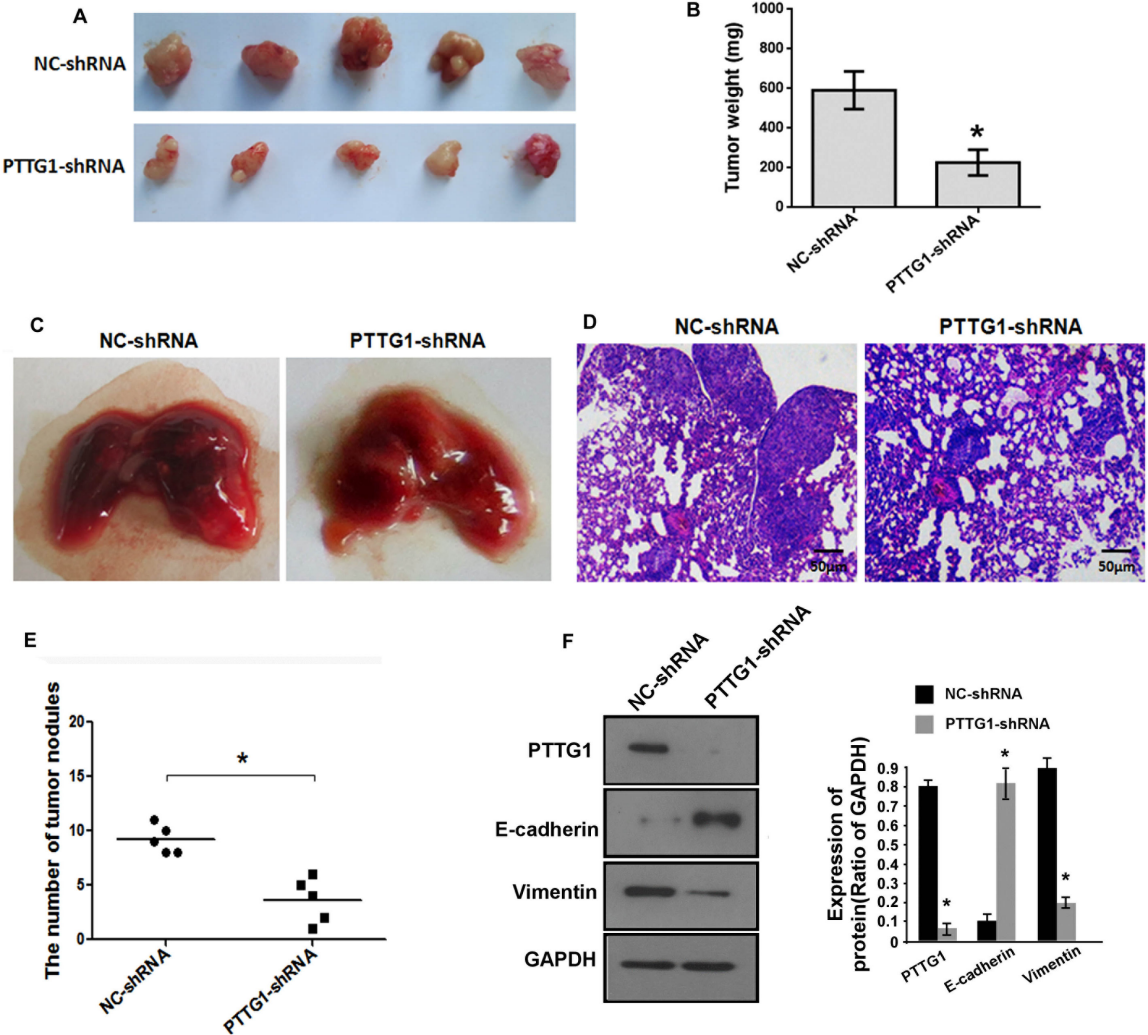
PTTG1 knockdown inhibited BC growth and metastasis *in vivo* (**A**) The representative images of xenograft tumor of PTTG1 knockdown groups and control groups. (**B**) PTTG1 knockdown significantly inhibited xenograft tumor weight. (**C**) The representative images of metastatic tumors in lung of PTTG1 knockdown groups and control groups. (**D**) HE analysis of the tumor in lung. Scale bar, 50 μm. (**E**) The number of tumor nodules were reduced after PTTG1 knockdown. (**F**) Western blot analyzed PTTG1, E-cadherin and Vimentin expression in xenograft tumor with PTTG1 knockdown. GAPDH was used as the loading control. *P < 0.05, ***P < 0.001. Results were the means ± SD in triplicate.

We performed *in vivo* growth and metastasis assay to confirm whether PTTG1 regulated BC growth and metastasis. Xenograft tumor analysis suggested PTTG1 knockdown inhibited tumor growth (Figure 3A), and significantly reduced tumor weight (Figure 3B). Lung metastasis *in vivo* assay suggested PTTG1 knockdown inhibited the lung metastasis of BC cell (Figure 3C). HE assay revealed that the metastatic tumors were reduced in lung suggesting PTTG1 knockdown inhibited lung metastasis (Figure 3D). The number of tumor nodules was also reduced compared to the control groups (Figure 3E). We also isolated the total protein of tumor, western blot assay suggested epithelial marker E-cadherin level was significantly increased, mesenchymal marker Vimentin was significantly reduced (Figure 3F), confirming PTTG1 regulated EMT and metastasis. Together, PTTG1 knockdown induced senescence and inhibited migration, invasion, metastasis and growth of BC cells.

### PTTG1 is negatively correlated with miR-146a-3p in BC tissues and cell lines

**Figure 4:**
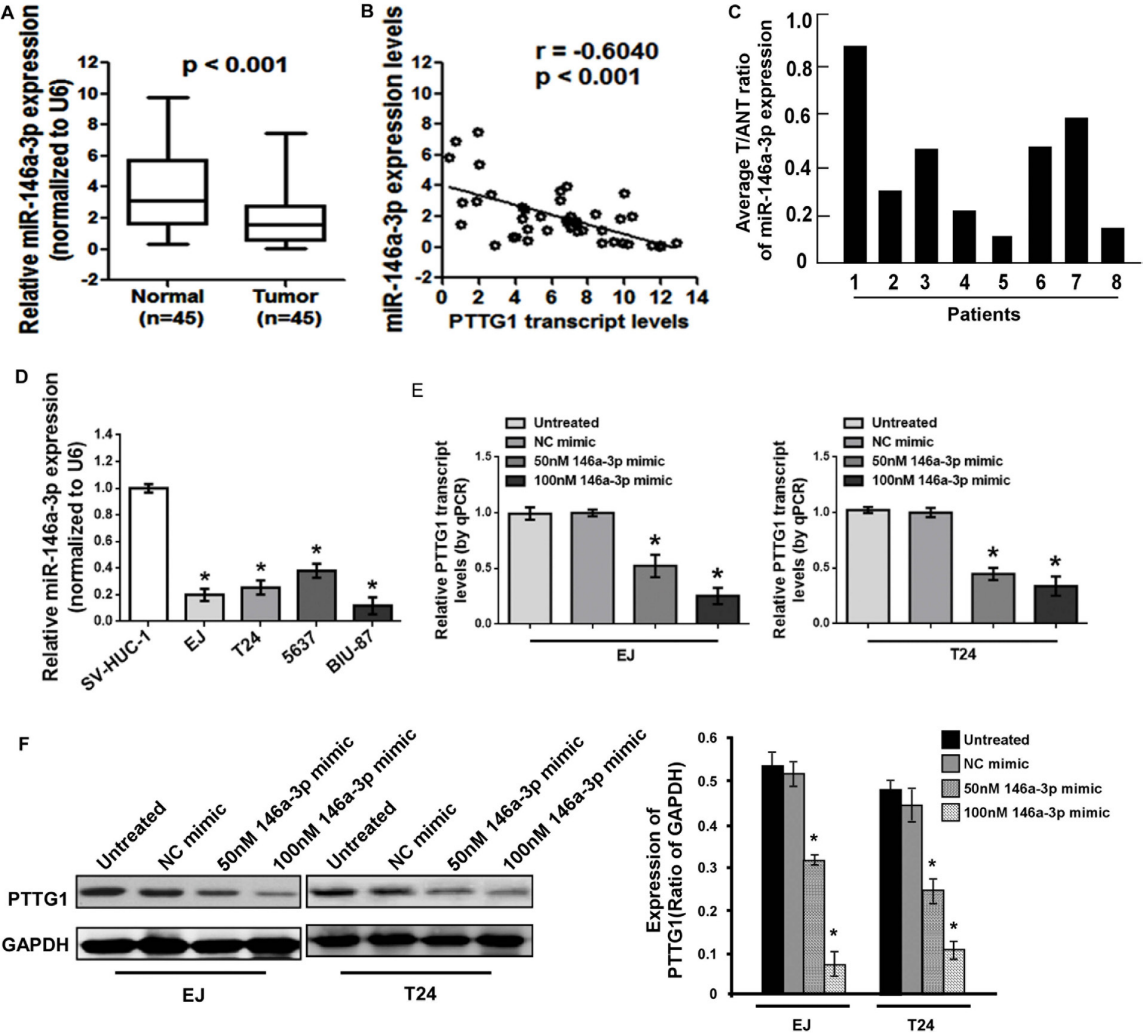
miR-146a-3p was downregulated in BC cells and tissues (**A**) Real-time RT-PCR analysis suggested miR-146a-3p was downregulated in BC tissues compared to the adjacent normal bladder tissues. (**B**) miR-146a-3p levels were negatively correlated with PTTG1 levels in BC tissues. (**C**) Real-time RT-PCR analysis suggested miR-146a-3p was downregulated in another eight BC tissues compared to the adjacent normal bladder tissues. (**D**) Real-time RT-PCR analysis suggested miR-146a-3p was downregulated in BC cells. (**E**) Real-time RT-PCR analysis suggested miR-146a-3p overexpression inhibited PTTG1 mRNA levels. (**F**) Western blot assay suggested miR-146a-3p overexpression decreased PTTG1 protein levels. GAPDH was used as the loading control. *P < 0.05. Results were the means ± SD in triplicate.

miRNAs could regulate target genes expression by inhibition mRNA translation and/or degradation. We used online software packages including Target-Scan, microRNA.org, miRanda and miRWalk to predict the miRNAs which could regulate PTTG1, and found miR-146a-3p might inhibit PTTG1. Real-time RT-PCR analysis showed that miR-146a-3p was significantly decreased in BC tissues compared to the adjacent normal bladder tissues (Figure 4A). The correlation analysis indicated miR-146a-3p levels had a negative correlation with PTTG1 levels in BC tissues (*P* < 0.001, r = −0.6040) (Figure 4B). We also analyzed miR-146a-3p levels in another eight BC tissues, and found miR-146a-3p was downregulated in BC tissues compared to the adjacent normal bladder tissues (Figure 4C). miR-146a-3p was also significantly downregulated in BC cells compared to the normal uroepithelium cell SV-HUC-1 (Figure 4D). To determine whether miR-146a-3p regulates the expression of PTTG1, we transfected with 50 nM or 100 nM miR-146a-3p mimic into EJ and T24, respectively. Real-time RT-PCR and Western blot assay showed that both mRNA and protein of PTTG1 were significantly downregulated in a concentration-dependent manner in both cell lines (Figure 4E and 4F). These findings suggested miR-146a-3p inhibited PTTG1 expression.

### miR-146a-3p directly targets the 3′UTR of PTTG1

**Figure 5:**
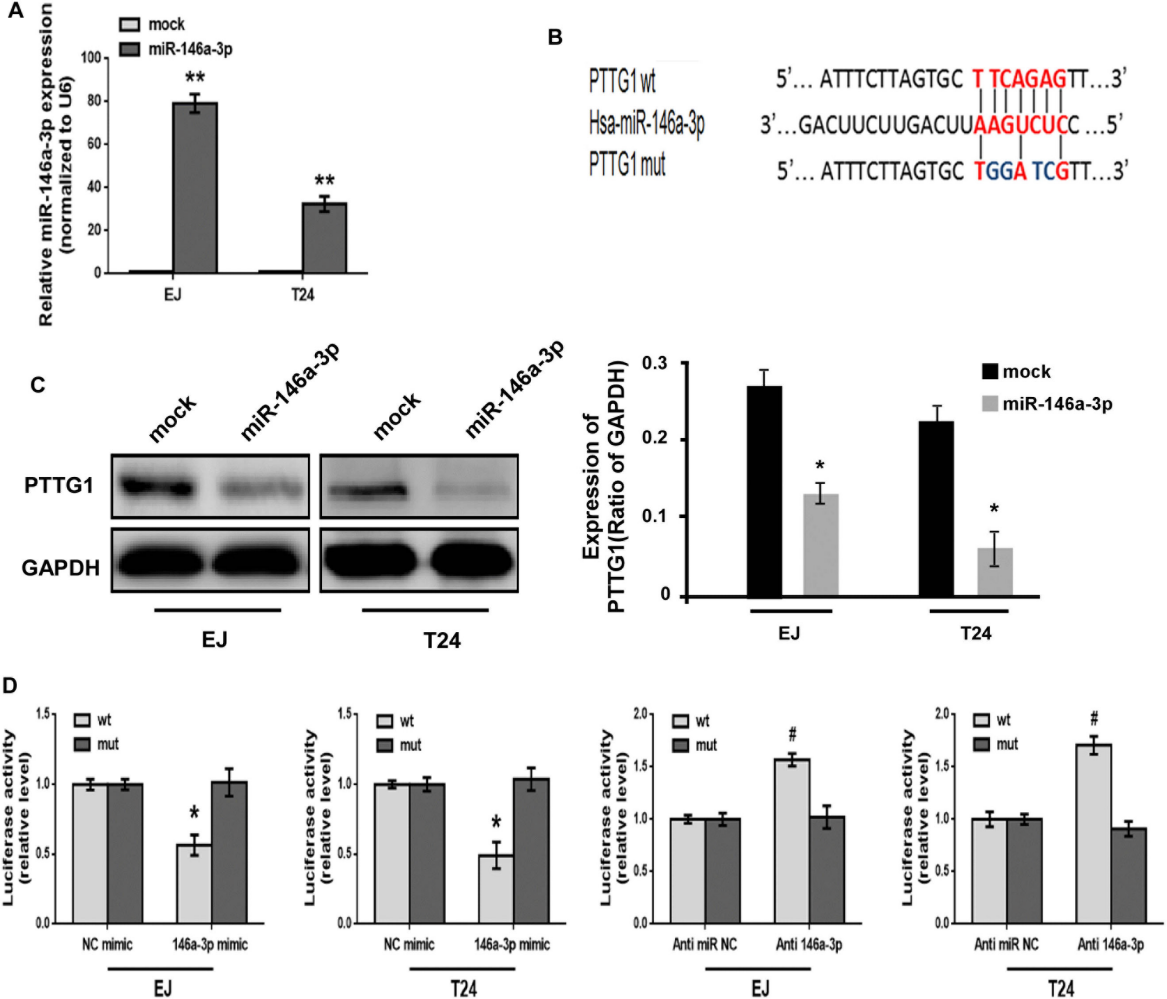
PTTG1 was the target of miR-146a-3p (**A**) Real-time RT-PCR analyzed miR-146a-3p expression after transfection with miR-146a-3p overexpression vector. (**B**) Schematic representation of miR-146a-3p target binding site in the 3′UTR of PTTG1. Wild type (wt) and mutated type (mut) of 3′UTR in seed sequences were indicated. (**C**) Western blot determined PTTG1 expression after miR-146a-3p overexpression. GAPDH was used as the loading control. (**D**) Luciferase reporter assay determined the luciferase activity after co-transfection with luciferase reporter vector containing wild or mutated 3′UTR of PTTG1 sequence and miR-146a-3p mimic or miR-146a-3p antagomirs. ^*/#^*P* < 0.05. Results were the means ± SD in triplicate.

To further investigate the function of miR-146a-3p in BC cells, we constructed miR-146a-3p overexpression vectors and transfected them into EJ and T24 cells for stable expression of miR-146a-3p. The expression of miR-146a-3p was confirmed to be significantly upregulated in miR-146a-3p-transduced EJ and T24 cells (Figure 5A). Bioinformatics analyses predicted that the 3′UTR of PTTG1 contain binding sites for miR-146a-3p (Figure 5B). Similar to above result, miR-146a-3p overexpression inhibited PTTG1 expression (Figure 5C). To determine whether miR-146a-3p represses PTTG1 expression by directly binding with the 3′UTR of PTTG1, luciferase reporter system assay suggested that the luciferase activity was significantly reduced after co-transfection with miR-146a-3p mimic and luciferase reporter vector containing 3′UTR of PTTG1, luciferase activity was significantly increased after co-transfection with miR-146a-3p antagomirs and luciferase reporter vector. However, luciferase activity didn't change after co-transfection with mutated luciferase reporter vector and miR-146a-3p mimic or miR-146a-3p antagomirs (Figure 5D), suggesting PTTG1 was the target of miR-146a-3p.

### miR-146a-3p overexpression suppresses BC cell migration, invasion and cell cycle progression and induces senescence in a PTTG1-dependent manner

**Figure 6:**
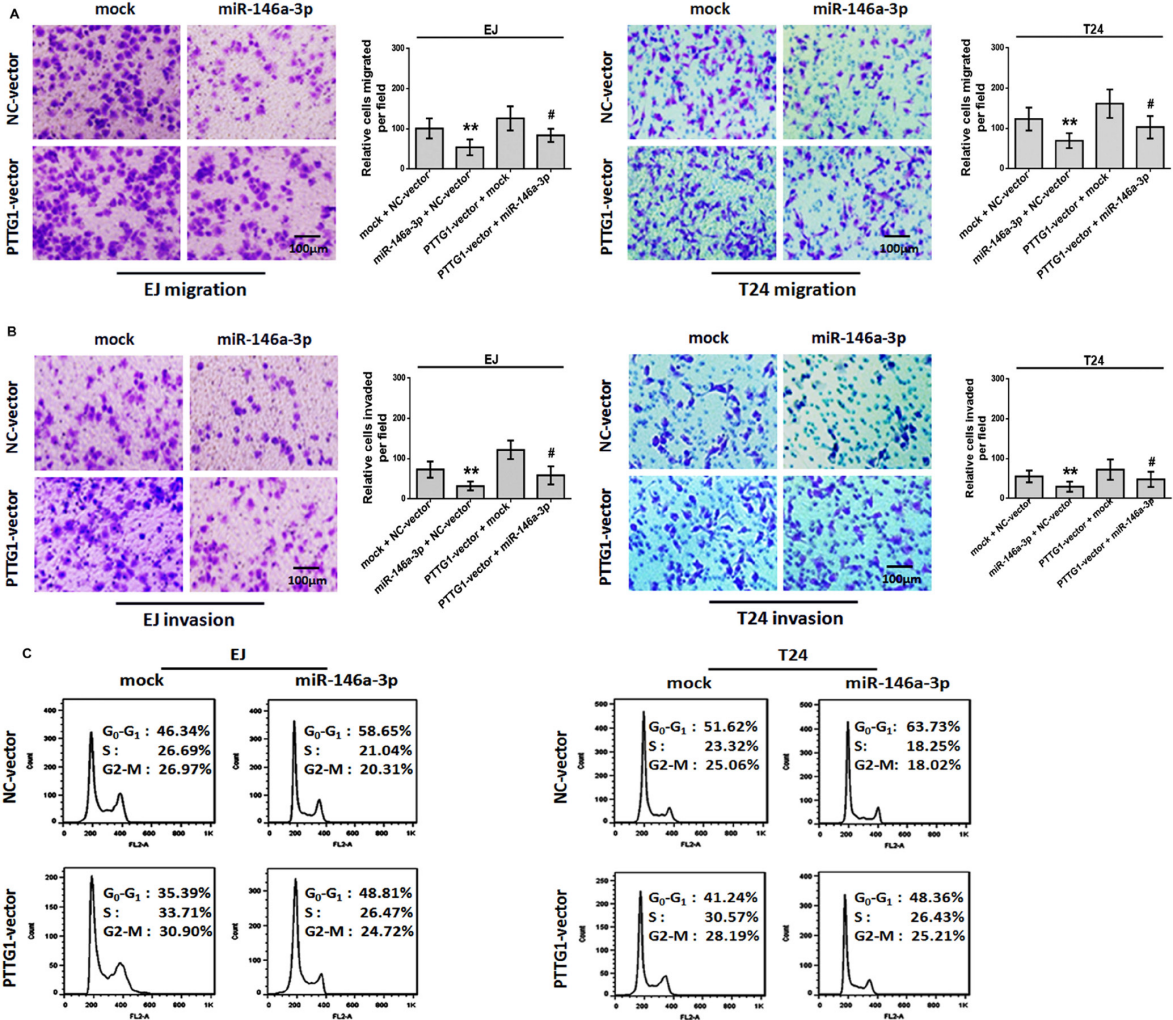
miR-146a-3p overexpression inhibited migration, invasion and cell cycle progression in a PTTG1-dependent manner (**A**–**B**). BC cells transfected with PTTG1 vector and miR-146a-3p alone and in combination as indicated and subjected to Transwell assay without (A) or with (B) Matrigel. Scale bar, 100 μm. (**C**) Flow cytometry assay of BC cells transfected with PTTG1 vector, miR-146a-3p alone or in combination as indicated. *P < 0.05. Results were the means ± SD in triplicate.

Given that PTTG1 was the target of miR-146a-3p, and promoted BC progression, we performed rescue experiment to confirm whether miR-146a-3p overexpression could reverse the phenotypes of PTTG1 overexpression. The transwell assay with or without matrigel suggested miR-146a-3p overexpression significantly inhibited BC cell migration and invasion, while PTTG1 overexpression promoted BC cell migration and invasion, miR-146a-3p and PTTG1 co-overexpression restored BC cell migration and invasion abilities compared to miR-146a-3p overexpression (Figure 6A and 6B). The flow cytometry assay revealed that overexpression of miR-146a-3p resulted in cell cycle arrest at G0/G1 phase. Meanwhile, re-expression of PTTG1 decreased the percentage of cells in G0/G1 phase and effectively reversed the miR-146a-3p overexpression induced

**Figure 7:**
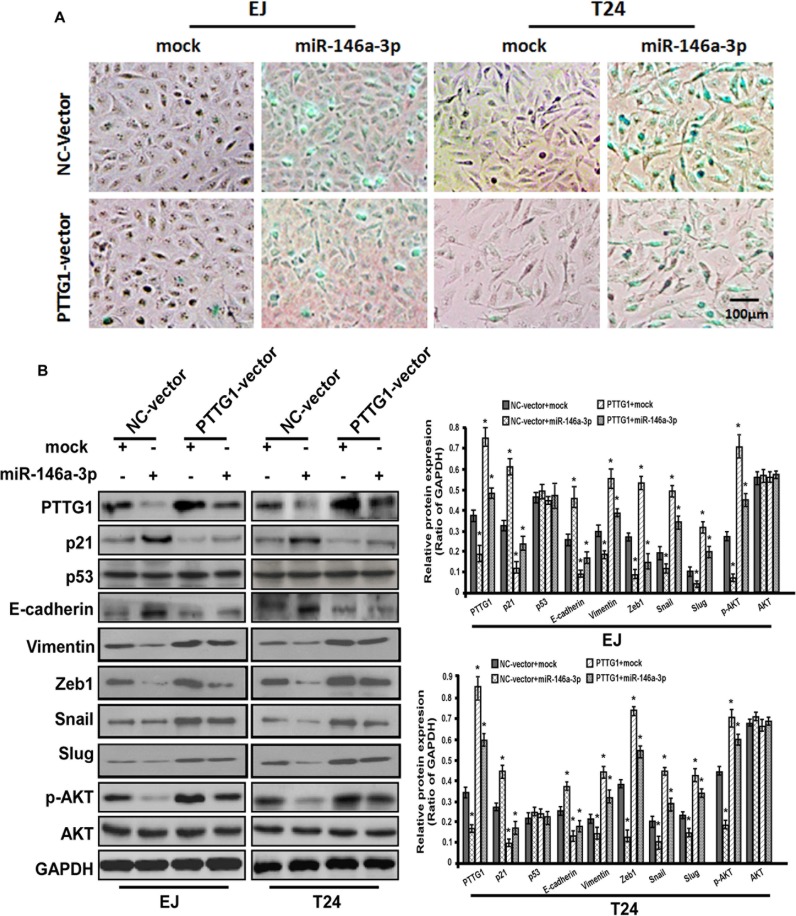
miR-146a-3p overexpression induced senescence in a PTTG1-dependent manner (**A**) SA-βgal activity assay suggested miR-146a-3p overexpression induced senescence, while PTTG1 co-overexpression abrogated this effect in BC cell. Scale bar, 100 μm. (**B**) Western blot assay studied p21, p53, E-cadherin, Vimentin, Zeb1, Snail, Slug, p-AKT and AKT expression after miR-146a-3p and PTTG1 overexpression alone and co-overexpression in BC cells, GAPDH was used as the loading control. *P < 0.05. Results were the means ± SD in triplicate.

G0/G1 phase arrest (Figure 6C). SA-βgal activity assay suggested miR-146a-3p overexpression induced senescence, miR-146a-3p and PTTG1 co-overexpression reversed miR-146a-3p overexpression induced senescence (Figure 7A). In addition, we examined the expressions of some key proteins regulated senescence, EMT, migration, invasion and metastasis. Western blot assay showed that the overexpression of miR-146a-3p decreased the levels of PTTG1, Zeb1, Vimentin, Snail, Slug and p-AKT and elevated the levels of p21 and E-cadherin, p53 and AKT weren't changed. Whereas the co-transfection with PTTG1 and miR-146a-3p overexpression vectors abrogated the effects of miR-146a-3p on the levels of these proteins (Figure 7B). These results suggested that miR-146a-3p affects the cell cycle, senescence, migration and invasion of BC cells in a PTTG1-dependent manner, confirming PTTG1 was the target of miR-146a-3p.

### miR-146a-3p inhibits the growth and metastasis *in vivo* of BC cells

**Figure 8:**
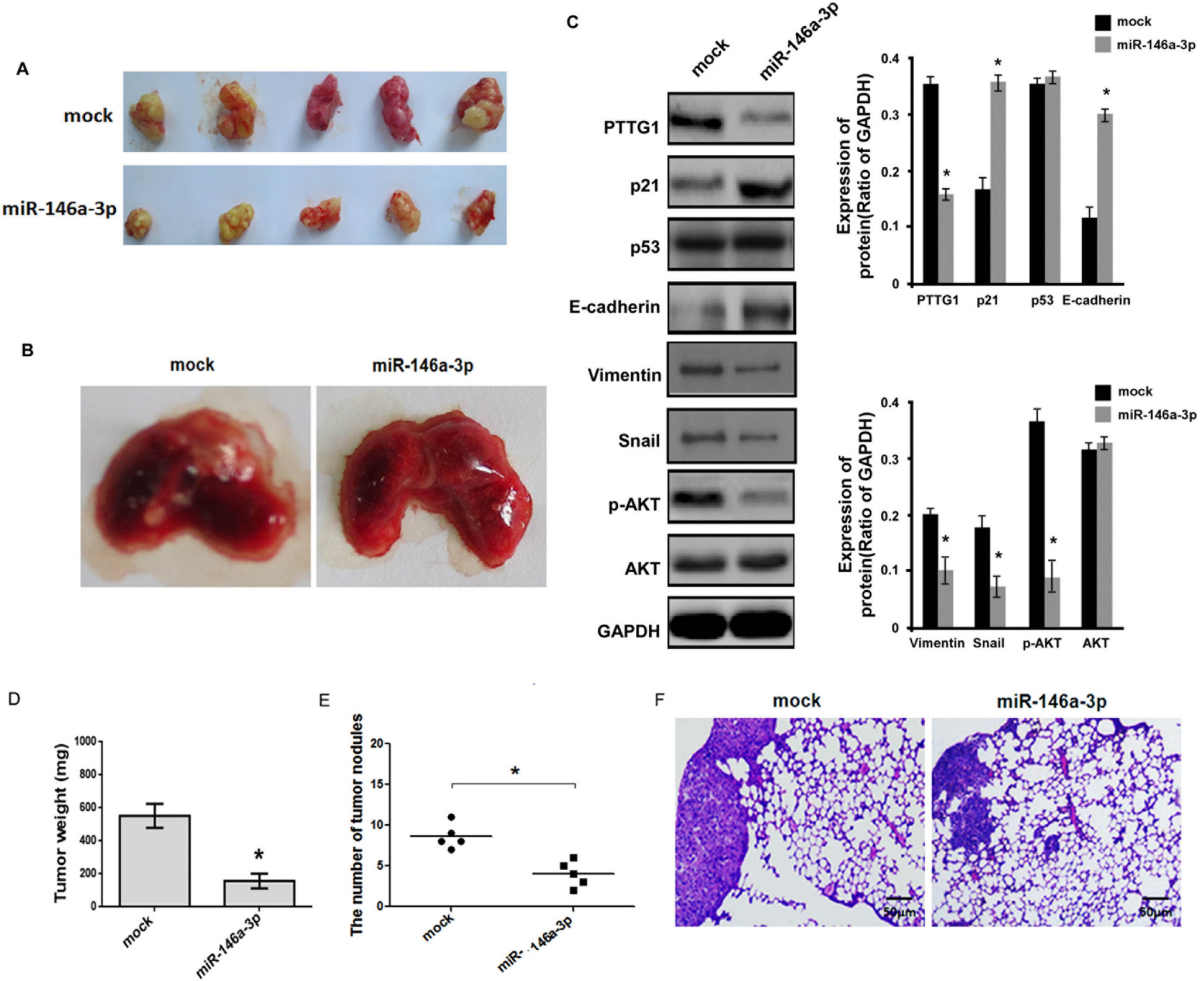
miR-146a-3p inhibited the growth and metastasis of BC cells *in vivo* (**A**) The representative images of xenograft tumor of miR-146a-3p overexpression groups and control groups. (**B**) The representative images of metastatic tumors in lung of miR-146a-3p overexpression groups and control groups. (**C**) Western blot analyzed PTTG1, p21, p53, E-cadherin, Vimentin, Snail, p-AKT and AKT expression in xenograft tumor with miR-146a-3p overexpression. GAPDH was used as the loading control. (**D**) miR-146a-3p overexpression significantly inhibited xenograft tumor weight. (**E**) The number of tumor nodules in lung was reduced after miR-146a-3p overexpression. (**F**) HE analysis of the tumor in lung. Scale bar, 50 μm. *P <0.05, **P < 0.01. Results were the means ± SD in triplicate.

Above study showed miR-146a-3p inhibited BC cell cycle progression, migration and invasion and induced senescence, we performed animal experiments to further demonstrate these results. miR-146a-3p overexpression inhibited tumor growth and lung metastasis (Figure 8A and 8B). Total protein of tumor tissues was isolated, western blot assay suggested miR-146a-3p overexpression inhibited PTTG1, Vimentin, Snail and p-AKT expression, and increased p21 and E-cadherin expression, p53 and AKT weren't changed (Figure 8C). Tumor weight and the number of tumor nodules were significantly decreased (Figure 8D and 8E). HE assay also suggested metastatic tumors were reduced in lung after miR-146a-3p overexpression (Figure 8F). These findings suggested miR-146a-3p overexpression inhibited BC growth and metastasis.

## DISCUSSION

In present study, we studied the role of PTTG1 in BC progression. PTTG1 was upregulated in human BC tissues and cells. There was a significant correlation between high PTTG1 expression and TNM stage, tumor size, invasion and metastasis of BC, suggesting PTTG1 might be a potential biomarker for BC progression. Functional assay suggested PTTG1 knockdown induced senescence and inhibited the migration, invasion, metastasis and growth of BC *in vitro* and *in vivo*. Mechanism analysis suggested PTTG1 was a target of miR-146a-3p, miR-146a-3p overexpression induced senescence, and inhibited the migration, invasion, metastasis and growth of BC. Rescue experiment revealed that overexpression of PTTG1 reversed the effect of miR-146-3p on senescence, migration, invasion, metastasis and growth of BC cells, suggesting miR-146a-3p inhibited BC progression through PTTG1.

miRNAs have been showed to play an important role in the regulation of gene expression at post transcriptional level. miR-146a is a tumor suppressor in a wide range of tumors including gastric cancer and castration-resistant prostate cancer [25–29]. Recent report showed that miR-146a inhibited BC growth by targeting IRAK-1 (interleukin-1 receptor-associated kinase 1) and TRAF6 (tumor necrosis factor receptor-associated factor 6) [25]. However, there are still some cases of growth inhibition of BC cells induced by miR-146a in an IRAK-1 or TRAF6-independent manner. In our study, miR-146a-3p was significantly downregulated and inversely associated with the levels of PTTG1 in BC tissues and cells. miR-146a-3p overexpression suppressed migration, invasion, metastasis and growth, and enhanced senescence of BC cells, these effects were abrogated by the re-expression of PTTG1, indicating miR-146a-3p acted as a repressor of BC cells in a PTTG1-dependent manner. Further studies confirmed that miR-146a-3p directly targeted PTTG1 via binding to the 3′UTR of PTTG1. These results demonstrated that PTTG1 was the target of miR-146a-3p.

p21 which is a cell cycle inhibitor arrests cell cycle progression, furthermore, p21 induces G1-phase arrest and apoptosis in human BC cells [30]. Besides, p21 is a target of PTTG1 in pituitary tumor cells [31–33]. In our study, we showed PTTG1 overexpression inhibited p21 expression, whereas miR-146a-3p overexpression promoted p21 expression in BC cells. We also observed that miR-146a-3p inhibited the cell cycle progression and tumor growth, and induced senescence in a PTTG1-dependent manner, suggesting p21 was an important target for miR-146a-3p and PTTG1 in BC progression. On the other hand, p21 is demonstrated to be induced through a p53-dependent or -independent pathway in response to DNA damage or oncogene expression [34–36]. PTTG1 also regulates p53 in some types of human tumors, such as lung cancer and breast cancer [37, 38]. So we investigated whether PTTG1 regulates p53 in BC cells. However, we did not observe any significant change in p53 protein level. This might because that the induction of p21 was associated with wild type but not mutant type of p53 expression, genetic alternations of p53 are frequently observed in BC cells (including EJ and T24 cell lines) [39–41]. Our data suggested miR-146a-3p regulating PTTG1 facilitated p21 induction, and inhibited tumor growth in a p53-independent manner.

E-cadherin, Zeb1, Vimentin, Snail and Slug have been proven to be a key role in tumor invasion and metastasis [42–44]. PTTG1 triggered EMT by decreasing E-cadherin expression in human ovarian cancer cells [12]. We found that E-cadherin was significantly upregulated, and Zeb1, Vimentin, Snail and Slug were significantly downregulated after the silencing of PTTG1 or restoration of miR-146a-3p, accompanied by the suppression of migration, invasion and metastasis of BC cells *in vitro* and *in vivo*. These suggested miR-146a-3p and PTTG1 also regulated EMT in BC. In addition, AKT, the key component of PI3K/AKT/mTOR signaling pathway, is verified to be frequently activated in numerous kinds of human cancers including bladder carcinoma [45–47]. A recent report points out that PTTG1 promotes migration and invasion of breast cancer cells through activation of AKT [14]. In our study, we have found that the p-AKT level was dramatically decreased as a response to PTTG1 silencing or miR-146a-3p overexpression in BC cells [14]. All together, these findings indicated that E-cadherin, Zeb1, Vimentin, Snail, Slug and AKT are the downstream factors of the axis of miR-146a-3p/PTTG1.

In summary, we confirmed the oncogenic function of PTTG1 in BC and demonstrated that PTTG1 is a target of miR-146a-3p. We further verified that miR-146a-3p modulated senescence, migration, invasion, metastasis and growth of BC cells in a PTTG1-dependent manner. Moreover, p21, E-cadherin, Zeb1, Vimentin, Snail, Slug and p-AKT were the downstream molecules of miR-146a-3p/PTTG1 pathway. Taken together, miR-146a-3p/PTTG1 regulated BC progression. Both miR-146a-3p and PTTG1 could be considered as two promising targets for bladder carcinoma treatment in the future.

## MATERIALS AND METHODS

### Cell lines and clinical samples

The human BC cell lines EJ and BIU-87 were obtained from the Type Culture Collection of Chinese Academy of Sciences. Human BC cell lines T24 and 5637 as well as human normal uroepithelium cell SV-HUC-1 was purchased from American Type Culture Collection (ATCC, USA). They were tested and authenticated for genotypes by DNA fingerprinting. These cell lines were passaged for less than 6 months after resuscitation, and no reauthorization was done. EJ, T24, BIU-87 and 5637 cells were maintained in RPMI-1640 medium (Gibco, USA) supplemented with 10% fetal bovine serum (HyClone, USA), penicillin (100 U/mL) and streptomycin (100 mg/mL). SV-HUC-1 cells were cultured in F-12K medium (Gibco, USA) with 10% fetal bovine serum (HyClone, USA), penicillin (100 U/mL) and streptomycin (100 mg/mL). These cells were maintained in a 37°C humidified incubator with 5% CO_2_. In addition, 45 primary BC samples and adjacent normal bladder tissues were collected from patients who had radical cystectomy between 2012 and 2014 at Urology Department of Wuhan Union Hospital. All specimens were immediately snap-frozen in liquid nitrogen after surgical removal. Histological and pathological diagnoses were confirmed by at least two experienced pathologists. All of our experiments were undertaken with the understanding and written consent of each subject. The study methodologies conformed to the standards set by the Declaration of Helsinki, and the study methodologies were approved by the ethics committee of Tongji Medical College of Huazhong University of Science and Technology.

### Real-time RT-PCR analysis

Total RNA was isolated from the specimens and cell lines using TRIzol reagent (Invitrogen, USA). For PTTG1 mRNA analysis, complementary DNA was synthesized by using the reverse transcription kit (RR047A, TaKaRa). For the detection of mature miRNAs, the RNA was reversely transcribed into cDNA by using the SYBR^®^ PrimeScript™ miRNA RT-PCR Kit (RR716, TaKaRa). SYBR^®^ Premix Ex Taq™ (Tli RNaseH Plus) kit (RR420A, TaKaRa) was then utilized for Real-time RT-PCR detection. The nucleotide sequences of the primers were as follows: PTTG1: 5′-CCCTCAAACAAAAACAGCCAAG-3′ (forward), 5′-GGCATCATCTGAGGCAGGAAC-3′ (reverse). GAPDH served as an internal control using primers 5′-TC AAGAAGGTGGTGAAGCAG-3′ (forward) and 5′-CG TCAAAGGTGGAGGAGTG-3′ (reverse). The specific premiers for the candidate miRNAs were purchased from RiboBio (RiboBio Co., Ltd., China), and U6 was used as an internal control for detection of miRNAs. All analyses were performed by using the StepOnePlus^T^MReal-Time RT-PCR System (Applied Biosystems, USA). The 2^−ΔΔCT^ method was employed to calculate the relative expression of each gene.

### RNA oligoribonucleotides and transfection

The miR-146a-3p mimics and control group (NC mimic) were also designed and synthesized by RiboBio. RiboBio still provided the synthesized antagomirs against miR-146a-3p (anti 146a-3p) and control group (anti-miR NC). EJ and T24 cells were transiently transfected with the foregoing RNA oligoribonucleotides, respectively. Transfection was performed using Lipofectamine 2000 (Invitrogen, USA) according to the manufacturer's protocol.

### Plasmids and luciferase reporter assay

A DNA fragment containing the miR-146a precursor with 300 bp flanking sequence of each side was cloned into GV252 plasmids (Genechem Co., China). The plasmids were transfected into EJ and T24 cells using Lipofectamine 2000 (Invitrogen, USA) following the protocol, and empty vectors of GV252 were transfected into the same cells and functioned as the negative control (mock). Stable transfectants were selected by using 400 μg/ml and 600 μg/ml neomycin for T24 and EJ cells, respectively. PTTG1 shRNA expression vectors containing the target sequence (5′-GACCCUGGAUGUUGAAUUG-3′) and an empty vector of GV248 were also procured from Genechem Co. Puromycin (0.2 μg/ml and 0.4 μg/ml) was used for stable selection of T24 and EJ cells, respectively. The complementary DNA (cDNA) sequence of PTTG1 was cloned into the *BamH*I-*Age*I sites of GV218 vectors (Genechem) and was confirmed by DNA sequencing. The premier set was 5′-ACGGATCCCGA TGGCTACTCTGATCTATGTT-3′ (forward) and 5′-GG ACCGGTAATTAAATATCTATGTCACAGCAAACA-3′ (reverse). Wild type or mutant human PTTG1 3′UTR fragments (bases 655–713, NM 004219) containing the Xhol-Notl restriction sites were created by chemical synthesis, respectively. These fragments were inserted into the cloning site of the psi-CHECK-2 vector (Promega, USA), which containing a dual-luciferase reporter. For the luciferase reporter assay, EJ and T24 cells were transiently co-transfected with the reporter vectors (wild type reporter vectors or mutant reporter vectors), and miRNA mimics (miR-146a-3p mimic or NC mimic), or antagomirs against miRNAs (anti-miR-146a-3p or anti-miRNA NC). The luciferase activity was measured after 24 transfection using the Dual-Luciferase Reporter Assay System (Promega, USA) according to the kit instructions.

### Western blot, hematoxylin-eosin (HE) staining and immunohistochemistry (IHC)

Total proteins were isolated by RIPA (Thermo Scientific, USA) from tissues and cell lines and subjected to western blotting analysis. Thirty micrograms of each lysate was electrophoresed in 10% SDS-PAGE gels, then transferred to PVDF membrane (Millipore, USA) and detected by using ECL kit (Beyotime Biotechnology, China). The anti-PTTG1 (ab79546) antibody and anti-p53 (ab28) antibody were procured from Abcam (Cambridge, UK). p21 (#2947), E-cadherin (#4065), Zeb1 (#3396), Vimentin (#3932), Snail (#3879), Slug (#9585), p-AKT (#9275), Akt (#9272) and GAPDH (D16H11) antibodies were all obtained from Cell Signaling Technology Inc. (Vebery, MA, USA). Horseradish peroxidase-conjugated anti-rabbit IgG, acting as the secondary antibody, was provided by Wuhan Boster Bio-engineering Co. Ltd., Wuhan, China. HE staining was employed to detect the lung metastases in rat models. Immunostaining was performed on BC tissue sections that had been previously confirmed for the pathological pattern by pathologists. The avidin-biotin-peroxidase method was performed to detect the expression and location of the target gene. The anti-PTTG1 primary antibody was used at a dilution of 1:100. An Olympus microscope was used to obtain images.

### Cell migration, invasion, cell cycle and senescence assay

The EJ and T24 cells stably transfected with NC-shRNA or PTTG1-shRNA vectors were harvested for cell migration, invasion, senescence and cell cycle analysis. Meanwhile, the EJ and T24 cells stably transfected with negative control group vectors or miR-146a-3p vectors (miR-146a-3p) were further co-transfected with the empty vector (NC-vector) or PTTG1 overexpressing vector (PTTG1-vector), respectively. These cells were also collected for the analysis of cell migration, invasion, senescence and cell cycle progression. The cell migration and invasion assays were performed as described previously [48]. The number of migrating or invading cells was counted in three randomly chosen fields under an inverted microscope. Flow cytometry assay was carried out according to the kit instructions (KeyGEN Biotech., China). Assay for senescence-associated beta-galactosidase (SA-βgal) activity was implemented according to the manufacturer's protocol using the SA-βgal kit (Beyotime Biotechnology, China). The senescent cells were photographed via microscope.

### *In vivo* growth and metastasis

To investigate the effects of PTTG1 silencing and miR-146a-3p restoration on bladder tumor growth and metastasis *in vivo*, 6 × 10^6^ EJ cells stably transfected with NC-shRNA, PTTG1-shRNA, mock or miR-146a-3p vectors were subcutaneously injected into the back and upper region of right armpit of 5-week-old female nude mice (*n* = 5), respectively. At the same time, 3 × 10^6^ EJ cells stably expressing NC-shRNA, PTTG1-shRNA, mock or miR-146a-3p vectors were injected intravenously into 5-week-old female nude mice (*n* = 5), respectively. Four weeks later, the mice with subcutaneous implantation were sacrificed and examined for tumor size and weight. And after another four weeks, the mice with intravenous injection of cancer cells were harvested and evaluated for the possible tumor nodules within lungs surface. All the mice were routinely fed basal rodent chow and water *ad libitum* under a 12 h light–dark cycle. All animal experiments were approved by the Animal Care Committee of Tongji Medical College, Huazhong University of Science and Technology, Wuhan, China.

### Statistical analysis

The statistical analysis was performed by using SPSS 18.0 software package (SPSS Inc., IL, USA). Data were expressed as mean ± the standard deviation (SD). Student's *t*-test was used to compare continuous variables between two groups. *Chi*-square test was conducted to compare the difference in IHC. And the Spearman correlation was employed to assess the correlation between miRNAs and PTTG1 expression in cancer specimens. Significance was set at *P* < 0. 05.

## References

[1] Jemal A, Bray F, Center MM, Ferlay J, Ward E, Forman D (2011). Global cancer statistics. CA Cancer J Clin.

[2] Sfakianos JP, Kim PH, Hakimi AA, Herr HW (2014). The effect of restaging transurethral resection on recurrence and progression rates in patients with nonmuscle invasive bladder cancer treated with intravesical bacillus Calmette-Guerin. J Urol.

[3] Pei L, Melmed S (1997). Isolation and characterization of a pituitary tumor-transforming gene (PTTG). Mol Endocrinol.

[4] Tfelt-Hansen J, Kanuparthi D, Chattopadhyay N (2006). The emerging role of pituitary tumor transforming gene in tumorigenesis. Clin Med Res.

[5] Chao JI, Hsu SH, Tsou TC (2006). Depletion of securin increases arsenite-induced chromosome instability and apoptosis via a p53-independent pathway. Toxicol Sci.

[6] Zhang X, Horwitz GA, Prezant TR, Valentini A, Nakashima M, Bronstein MD, Melmed S (1999). Structure, expression, and function of human pituitary tumor-transforming gene (PTTG). Mol Endocrinol.

[7] Kakar SS, Malik MT (2006). Suppression of lung cancer with siRNA targeting PTTG. Int J Oncol.

[8] Zhang ML, Lu S, Zheng SS (2008). Epigenetic changes of pituitary tumor-derived transforming gene 1 in pancreatic cancer. Hepatobiliary Pancreat Dis Int.

[9] Wondergem B, Zhang Z, Huang D, Ong CK, Koeman J, Hof DV, Petillo D, Ooi A, Anema J, Lane B, Kahnoski RJ, Furge KA, Teh BT (2012). Expression of the PTTG1 oncogene is associated with aggressive clear cell renal cell carcinoma. Cancer Res.

[10] Cao XL, Gao JP, Wang W, Xu Y, Shi HY, Zhang X (2012). Expression of pituitary tumor transforming gene 1 is an independent factor of poor prognosis in localized or locally advanced prostate cancer cases receiving hormone therapy. Asian Pac J Cancer Prev.

[11] Heaney AP, Singson R, McCabe CJ, Nelson V, Nakashima M, Melmed S (2000). Expression of pituitary-tumour transforming gene in colorectal tumours. Lancet.

[12] Shah PP, Kakar SS (2011). Pituitary tumor transforming gene induces epithelial to mesenchymal transition by regulation of Twist, Snail, Slug, and E-cadherin. Cancer Lett.

[13] Chesnokova V, Zonis S, Rubinek T, Yu R, Ben-Shlomo A, Kovacs K, Wawrowsky K, Melmed S (2007). Senescence mediates pituitary hypoplasia and restrains pituitary tumor growth. Cancer Res.

[14] Yoon CH, Kim MJ, Lee H, Kim RK, Lim EJ, Yoo KC, Lee GH, Cui YH, Oh YS, Gye MC, Lee YY, Park IC, An S (2012). PTTG1 oncogene promotes tumor malignancy via epithelial to mesenchymal transition and expansion of cancer stem cell population. J Biol Chem.

[15] Yu SY, Liu HF, Wang SP, Chang CC, Tsai CM, Chao JI (2013). Evidence of securin-mediated resistance to gefitinib-induced apoptosis in human cancer cells. Chem Biol Interact.

[16] Chiong E, Kesavan A, Mahendran R, Chan YH, Sng JH, Lim YK, Kamaraj R, Tan TM, Esuvaranathan K (2011). NRAMP1 and hGPX1 gene polymorphism and response to bacillus Calmette-Guerin therapy for bladder cancer. Eur Urol.

[17] Kompier LC, van der Aa MN, Lurkin I, Vermeij M, Kirkels WJ, Bangma CH, van der Kwast TH, Zwarthoff EC (2009). The development of multiple bladder tumour recurrences in relation to the FGFR3 mutation status of the primary tumour. J Pathol.

[18] Adams J, Williams SV, Aveyard JS, Knowles MA (2005). Loss of heterozygosity analysis and DNA copy number measurement on 8p in bladder cancer reveals two mechanisms of allelic loss. Cancer Res.

[19] Lauss M, Aine M, Sjodahl G, Veerla S, Patschan O, Gudjonsson S, Chebil G, Lovgren K, Ferno M, Mansson W, Liedberg F, Ringner M, Lindgren D (2012). DNA methylation analyses of urothelial carcinoma reveal distinct epigenetic subtypes and an association between gene copy number and methylation status. Epigenetics.

[20] Kanakis D, Kirches E, Mawrin C, Dietzmann K (2003). Promoter mutations are no major cause of PTTG overexpression in pituitary adenomas. Clin Endocrinol.

[21] Hidalgo M, Galan JJ, Saez C, Ferrero E, Castilla C, Ramirez-Lorca R, Pelaez P, Ruiz A, Japon MA, Royo JL (2008). Methylation alterations are not a major cause of PTTG1 misregulation. BMC Cancer.

[22] Bartel DP (2004). MicroRNAs: genomics, biogenesis, mechanism, and function. Cell.

[23] Esquela-Kerscher A, Slack FJ (2006). Oncomirs - microRNAs with a role in cancer. Nat Rev Cancer.

[24] John B, Enright AJ, Aravin A, Tuschl T, Sander C, Marks DS (2004). Human MicroRNA targets. PLoS Biol.

[25] Wang M, Chu H, Li P, Yuan L, Fu G, Ma L, Shi D, Zhong D, Tong N, Qin C, Yin C, Zhang Z (2012). Genetic variants in miRNAs predict bladder cancer risk and recurrence. Cancer Res.

[26] Yao Q, Cao Z, Tu C, Zhao Y, Liu H, Zhang S (2013). MicroRNA-146a acts as a metastasis suppressor in gastric cancer by targeting WASF2. Cancer Lett.

[27] Ali S, Ahmad A, Aboukameel A, Ahmed A, Bao B, Banerjee S, Philip PA, Sarkar FH (2014). Deregulation of miR-146a expression in a mouse model of pancreatic cancer affecting EGFR signaling. Cancer Lett.

[28] Sun Q, Zhao X, Liu X, Wang Y, Huang J, Jiang B, Chen Q, Yu J (2014). miR-146a functions as a tumor suppressor in prostate cancer by targeting Rac1. Prostate.

[29] Cornett AL, Lutz CS (2014). Regulation of COX-2 expression by miR-146a in lung cancer cells. RNA.

[30] Yang K, Zheng XY, Qin J, Wang YB, Bai Y, Mao QQ, Wan Q, Wu ZM, Xie LP (2008). Up-regulation of p21WAF1/Cip1 by saRNA induces G1-phase arrest and apoptosis in T24 human bladder cancer cells. Cancer Lett.

[31] Chesnokova V, Zonis S, Kovacs K, Ben-Shlomo A, Wawrowsky K, Bannykh S, Melmed S (2008). p21(Cip1) restrains pituitary tumor growth. Proc Natl Acad Sci U S A.

[32] Brugarolas J, Moberg K, Boyd SD, Taya Y, Jacks T, Lees JA (1999). Inhibition of cyclin-dependent kinase 2 by p21 is necessary for retinoblastoma protein-mediated G1 arrest after gamma-irradiation. Proc Natl Acad Sci U S A.

[33] Gartel AL, Tyner AL (1998). The growth-regulatory role of p21 (WAF1/CIP1). Prog Mol Subcell Biol.

[34] Rodriguez R, Meuth M (2006). Chk1 and p21 cooperate to prevent apoptosis during DNA replication fork stress. Mol Biol Cell.

[35] Kotoku N, Higashimoto K, Kurioka M, Arai M, Fukuda A, Sumii Y, Sowa Y, Sakai T, Kobayashi M (2014). Xylarianaphthol-1, a novel dinaphthofuran derivative, activates p21 promoter in a p53-independent manner. Bioorg Med Chem Lett.

[36] Li Q, Li J, Wen T, Zeng W, Peng C, Yan S, Tan J, Yang K, Liu S, Guo A, Zhang C, Su J, Jiang M (2014). Overexpression of HMGB1 in melanoma predicts patient survival and suppression of HMGB1 induces cell cycle arrest and senescence in association with p21 (Waf1/Cip1) up-regulation via a p53-independent, Sp1-dependent pathway. Oncotarget.

[37] Bernal JA, Luna R, Espina A, Lazaro I, Ramos-Morales F, Romero F, Arias C, Silva A, Tortolero M, Pintor-Toro JA (2002). Human securin interacts with p53 and modulates p53-mediated transcriptional activity and apoptosis. Nat Genet.

[38] Yu R, Heaney AP, Lu W, Chen J, Melmed S (2000). Pituitary tumor transforming gene causes aneuploidy and p53-dependent and p53-independent apoptosis. J Biol Chem.

[39] Grimm MO, Jurgens B, Schulz WA, Decken K, Makri D, Schmitz-Drager BJ (1995). Inactivation of tumor suppressor genes and deregulation of the c-myc gene in urothelial cancer cell lines. Urol Res.

[40] Rieger KM, Little AF, Swart JM, Kastrinakis WV, Fitzgerald JM, Hess DT, Libertino JA, Summerhayes IC (1995). Human bladder carcinoma cell lines as indicators of oncogenic change relevant to urothelial neoplastic progression. Br J Cancer.

[41] Cooper MJ, Haluschak JJ, Johnson D, Schwartz S, Morrison LJ, Lippa M, Hatzivassiliou G, Tan J (1994). p53 mutations in bladder carcinoma cell lines. Oncol Res.

[42] Bindels EM, Vermey M, van den Beemd R, Dinjens WN, Van Der Kwast TH (2000). E-cadherin promotes intraepithelial expansion of bladder carcinoma cells in an *in vitro* model of carcinoma *in situ*. Cancer Res.

[43] Du HF, Ou LP, Yang X, Song XD, Fan YR, Tan B, Luo CL, Wu XH (2014). A new PKCalpha/beta/TBX3/E-cadherin pathway is involved in PLCepsilon-regulated invasion and migration in human bladder cancer cells. Cell Signal.

[44] McConkey DJ, Choi W, Marquis L, Martin F, Williams MB, Shah J, Svatek R, Das A, Adam L, Kamat A, Siefker-Radtke A, Dinney C (2009). Role of epithelial-to-mesenchymal transition (EMT) in drug sensitivity and metastasis in bladder cancer. Cancer Metastasis Rev.

[45] Chun KH, Kosmeder JW, Sun S, Pezzuto JM, Lotan R, Hong WK, Lee HY (2003). Effects of deguelin on the phosphatidylinositol 3-kinase/Akt pathway and apoptosis in premalignant human bronchial epithelial cells. J Natl Cancer Inst.

[46] Hassan B, Akcakanat A, Holder AM, Meric-Bernstam F (2013). Targeting the PI3-kinase/Akt/mTOR signaling pathway. Surg Oncol Clin N Am.

[47] Calderaro J, Rebouissou S, de Koning L, Masmoudi A, Herault A, Dubois T, Maille P, Soyeux P, Sibony M, de la Taille A, Vordos D, Lebret T, Radvanyi F (2014). PI3K/AKT pathway activation in bladder carcinogenesis. Int J Cancer.

[48] Valster A, Tran NL, Nakada M, Berens ME, Chan AY, Symons M (2005). Cell migration and invasion assays. Methods.

